# Complete and Annotated Genome Sequence of the Sourdough Lactic Acid Bacterium Lactobacillus fermentum IMDO 130101

**DOI:** 10.1128/genomeA.00256-18

**Published:** 2018-05-10

**Authors:** Marko Verce, Luc De Vuyst, Stefan Weckx

**Affiliations:** a Faculty of Sciences and Bioengineering Sciences, Research Group of Industrial Microbiology and Food Biotechnology (IMDO), Vrije Universiteit Brussel, Brussels, Belgium

## Abstract

Lactobacillus fermentum is a species of lactic acid bacteria that is frequently found in sourdough, a fermented flour-water mixture used in the production of bread and other baked goods. Here, we present the complete genome sequence of *L. fermentum* IMDO 130101, a candidate sourdough starter culture strain isolated from a backslopped rye sourdough.

## GENOME ANNOUNCEMENT

Sourdough is a mixture of flour and water that is fermented by yeasts and lactic acid bacteria (LAB) and acidifies the bread dough, provides it with leavening capacity, and modifies its flavor (1, 2). Dough acidification, due to lactic acid and acetic acid produced through heterolactic fermentation by LAB, imposes a major stress factor on the microorganisms present in sourdough, hence retarding the growth of spoilage microorganisms and enabling the proliferation of well-adapted, niche-specific microorganisms. A wide variety of LAB species often prevails in sourdoughs, including the obligately heterofermentative Lactobacillus fermentum. Lactobacillus fermentum IMDO 130101 was originally isolated from a rye sourdough and backslopped in the laboratory, and it showed the ability to ferment maltose, convert fructose into mannitol, tolerate acidic conditions down to pH 3.0, and use the arginine deiminase (ADI) pathway, underlining its potential as candidate sourdough starter culture strain (3–7).

Lactobacillus fermentum IMDO 130101 was sequenced and its genome was annotated to investigate the strain's metabolic potential at gene level and adaptations to the sourdough environment. To this end, DNA was extracted from *L. fermentum* IMDO 130101 cell pellets with the High Pure PCR template preparation kit, followed by RNase treatment and then purification with the High Pure PCR product purification kit. Sequencing was performed by the VIB Nucleomics Core Facility (Leuven, Belgium) using an 8-kb paired-end library and 454 pyrosequencing, as described previously (8). Reads were assembled using the GS De Novo Assembler v2.7 with default parameters, followed by gap closure with PCR and Sanger sequencing of the resulting amplicons (VIB Genetic Service Facility, Antwerp, Belgium) based on primers designed using Consed v23.0 (9). The starting point of the closed circular genome was repositioned to match the starting point of a representative genome of *L. fermentum* to facilitate further comparative genomics. The complete genome was automatically annotated using GenDB v2.2 (10), followed by extensive manual curation. Coding DNA sequences (CDSs) shorter than 150 nucleotides and without a functional annotation were omitted. Based on annotation evidence, some CDSs were reannotated as pseudogenes. The complete genome of *L. fermentum* IMDO 130101 consisted of one circular chromosome of 2,089,202 bp, with a GC content of 51.5%, and contained no plasmids. The almost equal lengths of the two replichores (1,043,104 bp and 1,046,092 bp) indicated the absence of large-scale assembly errors. The complete genome contained 2,032 predicted genes or, more precisely, 1,916 protein-encoding genes, 58 tRNA genes, 5 rRNA gene clusters, and 40 pseudogenes. Genome sequencing of the strain revealed the genetic basis for some of its sourdough-specific characteristics, including genes enabling maltose consumption through heterolactic fermentation, mannitol production from fructose, and arginine deiminase activity. Comparison with other *L. fermentum* strains will facilitate the search for additional adaptations to the sourdough environment.

### Accession number(s).

The complete annotated genome sequence has been deposited in ENA under the accession number LT906621.
